# Impaired Firing Behavior of Individually Tracked Paretic Motor Units During Fatiguing Contractions of the Dorsiflexors and Functional Implications Post Stroke

**DOI:** 10.3389/fneur.2020.540893

**Published:** 2020-10-29

**Authors:** Francesco Negro, Kathleen E. Bathon, Jennifer N. Nguyen, Cassidy G. Bannon, Claudio Orizio, Sandra K. Hunter, Allison S. Hyngstrom

**Affiliations:** ^1^Department of Clinical and Experimental Sciences, Research Center for Neuromuscular Function and Adapted Physical Activity “Teresa Camplani”, Università degli Studi di Brescia, Brescia, Italy; ^2^Uniformed Services, University of Health Sciences, Bethesda, MD, United States; ^3^Department of Physical Medicine and Rehabilitation, Medical College of Wisconsin, Milwaukee, WI, United States; ^4^Department of Physical Therapy, Marquette University, Milwaukee, WI, United States

**Keywords:** motor unit (MU), stroke, decomposition, fatigue, tracking, tibialis anterior, exercise, rehabilitation

## Abstract

**Introduction:** This study quantified stroke-related changes in the following: (1) the averaged discharge rate of motor units (individually tracked and untracked) identified from high-density electromyography (HD-EMG) recordings, (2) global muscle EMG properties of the dorsiflexors during a fatiguing contraction, and the relationship between task endurance and measures of leg function.

**Methods:** Ten individuals with chronic stroke performed a sustained sub-maximal, isometric, fatiguing dorsiflexion contraction in paretic and non-paretic legs. Motor-unit firing behavior, task duration, maximal voluntary contraction strength (MVC), and clinical measures of leg function were obtained.

**Results:** Compared to the non-paretic leg, the paretic leg task duration was shorter, and there was a larger exercise-related reduction in motor unit global rates, individually tracked discharge rates, and overall magnitude of EMG. Task duration of the paretic leg was more predictive of walking speed and lower extremity Fugl-Meyer scores compared to the non-paretic leg.

**Discussion:** Paretic leg muscle fatigability is increased post stroke. It is characterized by impaired rate coding and recruitment and relates to measures of motor function.

## Introduction

Impaired rate modulation and recruitment of motor units in chronic stroke survivors during sub-maximal fatiguing contractions of the leg muscles may limit functional endurance and interfere with mobility. Consistent with this notion, stroke survivors demonstrate altered kinematics after short bouts of walking (1–3) and decreased distance walked during the 6-min walk test compared with healthy controls (4). In addition, for a given level of muscle fatigue, there are greater detrimental effects on walking speed in chronic stroke survivors as compared to controls (5). Neuromuscular fatigability can be quantified as the acute exercise-induced reduction in force or the time able to maintain a force during a contraction (6). Neuromuscular fatigue can be due to the nervous system's inability to excite the muscle (i.e., central factors) or impairments in muscle contractile properties (6–8). Likely due to damage to motor centers following stroke, evidence suggests that central factors contribute more toward neuromuscular fatigability post stroke vs. exercise-induced changes in contractile function of the muscle (9, 10). If the central nervous system is unable to excite motor neuron pools to meet the force demands of the task, this would present as impaired rate coding and recruitment of motor units.

Previous work has documented reduced rate coding and recruitment during brief sub-maximal contractions (11, 12), but little is known about motor unit firing behavior during fatiguing contractions. Mcmanus et al. have recently shown that the mean discharge rate from populations of motor units in the paretic and non-paretic FDI at the end of fatiguing contraction was decreased compared to baseline levels (10). However, population discharge rates could reflect the recruitment and identification of different motor units at the end of the fatiguing contraction than at baseline—leaving it difficult to interpret changes in rate coding within a specific unit. In fact, previous studies investigating motor unit behavior during neuromuscular fatigue in stroke individuals have quantified the global behavior of subpopulations of motor units decomposed using intramuscular or surface electromyography (EMG) signals before and after a fatiguing task. Due to the relative large variability in the interference EMG signals and the low stability of standard EMG recordings, there is no guarantee that the same populations of motor units can be identified before and after a fatiguing task, limiting our ability to compare motor unit behavior. On the other hand, the use of high-density surface EMG decomposition can provide the possibility to track the behavior of the same motor units comparing their two-dimensional spatial representation before and after different interventions, overcoming the limitations of standard population measures (13).

For these reasons, no studies have quantified the discharge rate behavior of individually tracked motor units or global muscle activation of functionally relevant leg muscles such as the ankle dorsiflexor muscles over the course of fatiguing task. The ankle dorsiflexor muscles are necessary for toe clearance during the swing phase of gait (14), and volitional dorsiflexion is often decreased following a stroke (15). Therefore, understanding the modifications in the neural control of the dorsiflexors during a fatiguing task is important to predict motor behavior and gait performance in people with stroke.

The purpose of this study was to quantify stroke-related changes in (1) individually tracked and untracked motor unit discharge rates, (2) global muscle EMG variables of the dorsiflexors during a fatiguing contraction, and (3) relate task endurance to measures of leg function.

## Methods

All protocols were approved by the Institutional Review Board at Marquette University (HR-2753).

Ten individuals with chronic stroke (>6 months) participated in this study, five males and five females (see Table 1 for characteristics). Dorsiflexion force generated was measured by a load cell (SSM-AJ-150, Scottsdale, AZ) embedded in a custom-built ankle brace. Prior to acquisition, torque signals were low-pass filtered (500 Hz) and then sampled at 1 kHz using a data acquisition card (National Instruments Corp., Austin, TX) and PC. A constant current stimulator (Digitimer DS7AH, Welwyn Garden City, UK) delivered a rectangular pulse of 100-μs duration with a maximal amplitude of 400 V. The stimulation intensity (between 200 and 500 mA) was set to 10% above resting maximal twitch. Resting twitch measurements were performed by delivering electrical stimulation to the common peroneal nerve before and after the maximal voluntary contraction strength (MVC) trials during the baseline measures. Additionally, a resting twitch was elicited at the end of the fatigue task, after the MVC. The peak of the twitch torque was used as a measure of the contractile properties of the tibialis anterior muscle. Custom-written LabVIEW (National Instruments, Austin, TX) programs were used to generate stimulator and acquire all data. Peak and percent decline in MVC and resting twitch torque values in response to the fatigue protocol were calculated.

**Table 1:** Characteristics of the study participants.

**Subject ID**	**Age (years)**	**Years Post Stroke**	**LE-FM**
101	80–85	8	32
102	55–60	33	21
103	60–65	24	13
104	35–40	10	26
105	40–45	10	28
106	70–75	8	19
107	65–70	28	23
108	55–60	6	21
109	70–75	6	32
110	60–70	25	32
Average ± SD	60.4 ± 13	15.8 ± 10	24.7 ± 6

### Surface EMG

**Figure 1:**
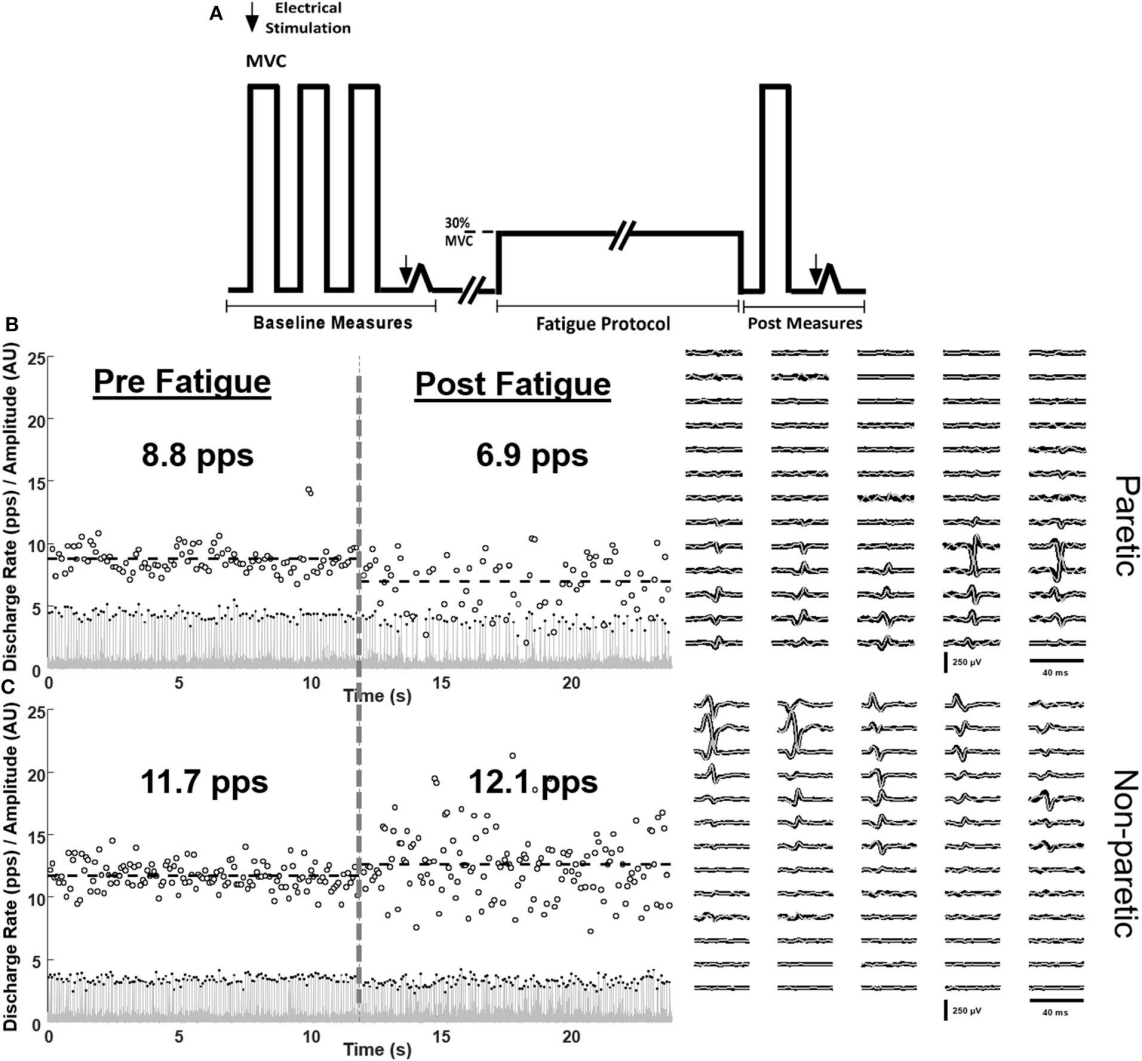
Schematic of the protocol **(A)** and single-subject examples of matched motor unit discharge rates of the paretic **(B)** and non-paretic **(C)** tibialis anterior muscle at baseline (left) and in response to fatiguing contractions (right). Innervation pulse trains (gray lines), identified spikes (black dots), instantaneous discharge rates (empty circles), and the spike triggered averaged motor unit action potentials of the tracked motor units pre (black) and post (gray) fatigue are shown.

One 64-channel surface matrix was positioned over the tibialis anterior muscle. The high-density surface EMG matrix was placed with the center at 1/3 on the line between the tip of the fibula and the tip of the medial malleolus. The monopolar surface EMG signals were amplified (EMG-USB2+, OT Bioelettronica, Italy), band-pass filtered (20–500 Hz), sampled at 2,048 Hz, and synchronized with the force signal. The EMG signals were decomposed into series of motor unit (MU) discharges using a convolutive blind source separation method (13, 16). This algorithm and similar blind source separation approaches have been previously validated and guarantee high accuracy in the identification of MU discharge times (17–20). The decomposition accuracy was estimated with the silhouette measure (SIL), with an acceptable threshold of 0.87. The individual motor units were decomposed independently at the beginning and end of the fatiguing task and, in a subset of subjects, tracked across the two-time segments by two-dimensional cross-correlation (13, 21, 22). Matched motor units were identified by a normalized 2D cross-correlation >0.80. The threshold for the 2D cross-correlation was selected based on previous works that have shown a good reliability in matching motor units across different recording sessions using such level of similarity. From the decomposed discharge times, the average discharge rate and the coefficient of variation for inter-spike intervals of the individual motor units were computed. An example of motor unit matching is shown in Figures 1B,C for the paretic and non-paretic leg of one subject. The averaged root mean square (RMS) of EMG across the grid was calculated for 10 s at baseline and at the end of the fatigue protocol (at 5–10 s steady state prior to meeting task failure criteria). The percent change in RMS EMG from fatigue to baseline was calculated. Discharge statistics of the identified motor units was calculated in the same segments.

### Protocol

Subjects were positioned in supine on a therapy table with their test leg positioned (ankle 35°s of plantarflexion, knee neutral, hip 10–15°s of flexion) in a custom-built ankle and lower leg brace that was secured to the table with Velcro straps. With visual feedback, 3–5 isometric dorsiflexor MVCs were made and immediately followed by a resting twitch measurement (Figure 1A). Resting twitch measurements were performed by delivering electrical stimulation to the tibialis anterior muscle at rest. Next, subjects were instructed to generate and sustain a target force of 30% of their MVC. The level of force was selected in order to understand the neuromechanical manifestations of fatigue during a task at moderate effort, similar to everyday life tasks. Tasks at higher force levels would lead to very short time-to-task failure with minimal influence of peripheral muscle properties. Criteria for task failure were as follows: failure to maintain target force for five consecutive seconds or five deviations below the target torque within a 10-s window. An error window of 10% of the target level was used. Upon meeting the task failure criteria, a final MVC and resting twitch measurement was performed. After a period of at least 15 min, the contralateral leg was tested (order counterbalanced). On a separate day, clinical measures of the LE-Fugl-Meyer and Ten Meter Walk Test were performed by a licensed physical therapist blinded to the fatigue testing.

Data are reported as mean ± standard deviation (±SD) and α = 0.05. Normal distribution of the analyzed variables was verified using the Shapiro–Wilk test. Separate paired *t*-tests were performed on the following variables: MVC, task duration, percent decline in MVC, and percent increase in RMS of EMG. A student's *t*-test was performed to detect differences with the percent change in discharge rates for the matched units. A mixed-model ANOVA was used for the population discharge rate analysis.

## Results

### Force and Task Duration Measurements

The paretic dorsiflexor isometric MVC force was less than that of the non-paretic MVC (106 ± 54 N vs. 177 ± 114 N, *P* < 0.01). Task duration was less for the paretic leg compared with the non-paretic leg (297.9 ± 217 s vs. 524.9 ± 262 s, *P* = 0.04). The relative decrease in MVC force due to the fatigue protocol was similar between the paretic and non-paretic legs (38 ± 15% vs. 36 ± 9%, *P* = 0.59). There was a trend for the relative decrease in resting twitch torque to be greater for the non-paretic than the paretic leg (55 ± 24% vs. 36 ± 33%, *P* = 0.05).

### Motor Unit Rate Coding and Recruitment in Response to Fatiguing Exercise

**Figure 2:**
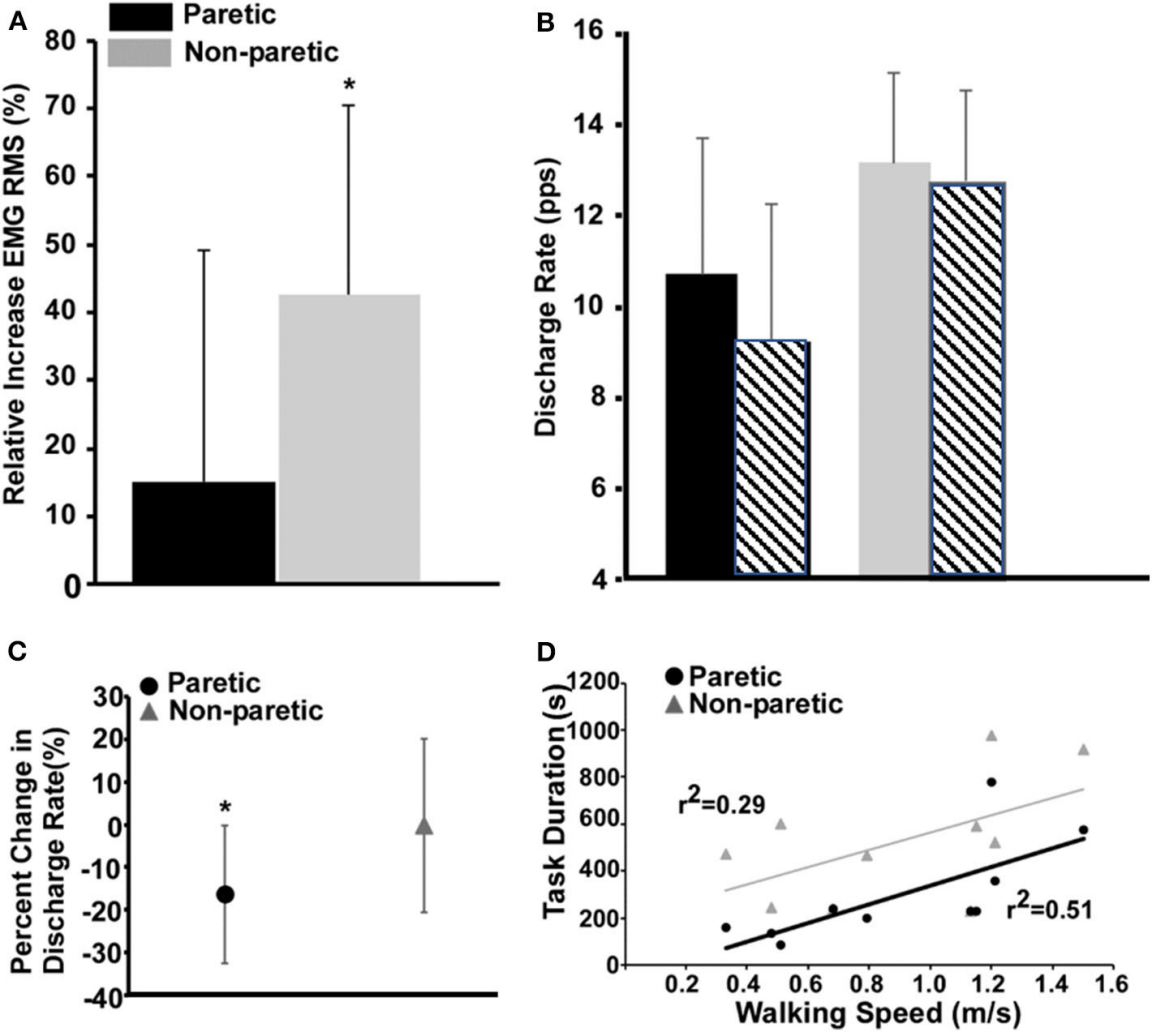
Measures of fatigue-related differences in the paretic and non-paretic tibialis anterior RMS EMG **(A)**, population discharge rates **(B)**, individually tracked discharge rates **(C)**, and the relationship between task duration and walking speed **(D)**. **(A)** Values for the paretic leg are shown in black and the ones for the non-paretic in gray. **(B)** Same as **(A)**, and the striped bars show the corresponding values after the fatigue task. **(C)** The circle and the triangle depict the variations in discharge rates of the matched motor units for the paretic and non-paretic leg, respectively. **(D)** Comparison of the regressions between task duration (s) and walking speed (m/s) for paretic and non-paretic sides.

The non-paretic leg had a larger relative increase in the global RMS magnitude of the EMG compared with the paretic leg (Figure 2A, *P* = 0.02). In total, 582 motor units were reliably decomposed (paretic leg: 162 pre fatigue and 138 post fatigue; non-paretic leg: 153 pre fatigue and 129 post fatigue), with an average of 16 ± 2 per subject in each leg/condition. A representative example of decomposition and matching is shown in Figures 1B,C. With respect to global measures of motor unit discharge rates (Figure 2B), there was a main effect of leg (p < np, *P* < 0.01) and time (post fatigue < pre fatigue, *P* < 0.01) and an interaction effect where there was a larger reduction in paretic leg discharge rates as compared to the non-paretic leg (*P* = 0.023). Similarly, in the subset of matched motor units identified in six subjects (90 motor units, 58 in paretic and 32 in the non-paretic leg), the paretic leg had a greater relative decrease in discharge rates compared with the non-paretic leg (Figure 2C, *P* < 0.001). Coefficient of variation for inter-spike intervals was greater post fatigue (*P* = 0.01), but no effect of legs was found (*P* = 0.34).

### Relationship Between Task Duration and Leg Function

Task duration of the paretic leg was positively correlated with walking speed (Figure 2D, *P* = 0.02) and the lower extremity Fugl-Meyer score (*r*^2^ = 0.54, *P* = 0.01). Task duration of the non-paretic leg was not significantly correlated with walking speed (*P* = 0.11).

## Discussion

As compared to the non-paretic leg, we show a decreased task endurance of the paretic dorsiflexors during a sub-maximal fatiguing contraction with greater reductions in the discharge rates of individually tracked and untracked motor units and global measures of whole muscle activation (Figure 2). These results are accompanied by, on average, a limited reduction in resting twitch response as compared to the non-paretic leg and comparably decreases in MVC. Finally, we are the first to establish a positive relationship between a measure of fatigability, task duration, and walking speed and the Fugl-Meyer score. Taken together, these results suggest that during fatiguing contractions of the paretic leg, the nervous system is unable to modulate rate coding or recruitment subsequently leading to the inability to maintain a sub-maximal force.

Here, we show deficiencies in the neural modulation of individual and populations of motor units during fatiguing contractions of the tibialis anterior, a muscle critical for walking function. Our evidence of a strong central (vs. peripheral) contribution to fatigue is consistent with previous investigations of mechanisms of neuromuscular fatigability post stroke. However, these studies focused on global measures of neural drive and excitability of the entire pathway from the cortex to muscle output (23–26) or, as with McManus et al., measures of discharge rates of populations of motor units in small hand muscles (10). Similar to the McManus study, during fatigue we showed a decrease in paretic motor unit discharge rates compared with the non-paretic muscle. However, in contrast, we showed a nearly 2-fold difference in task duration between the paretic and non-paretic muscles whereas the McManus study reported similar task durations. This study differences may be due to either (1) differences in fatigability of lower vs. upper limb muscle fatigability or (2) differences in the absolute force levels generated during the contractions. Neural drive to muscle (RMS of EMG and averaged discharge rate of motor units) typically increases during sub-maximal low- to moderate-intensity fatiguing contractions in healthy individuals (27–35). Thus, our findings suggest that decreased rate coding and or the drop out of motor units likely contributed to the reduced magnitude of the paretic RMS EMG compared to the non-paretic leg and is consistent with findings from other studies showing impaired ability to modulate EMG post stroke and different load levels and during fatigue (36, 37). Our data demonstrate that the inability of the nervous system to activate individual units, populations of units, and the whole muscle at baseline is exacerbated with ongoing muscle contractions in the paretic leg of stroke individuals.

Additionally, in our study, we found a positive correlation between task duration of the paretic leg's tibialis anterior and walking speed and no significant correlation to walking speed with the non-paretic leg's task duration. Although not directly tested, these data suggest that impairments in the paretic leg are more predictive of walking function vs. the non-paretic leg. This is consistent with other studies which correlate strength of the paretic leg and walking speed (38–40). Previously, we have shown that fatigue-related reductions in maximal paretic hip flexor contraction strength were correlated with baseline walking speed (41) and dynamic fatiguing contractions of the paretic hip flexors caused a larger decrease in walking speed as compared to controls (5). To our knowledge, the present study is the first to show the relationship between neuromuscular fatigability of the ankle dorsiflexors and baseline walking performance in chronic stroke.

We should acknowledge some limitations of the study design of our study: (1) the enrolled chronic stroke patients had a relatively large difference in the years post stroke and LE-FMA scores and this may have increased the variability of the outcome measures. (2) The fatigue protocol was performed on both legs on the same day, including a recovery time between recordings based on the perceived fatigue of the subjects. For this reason, we cannot exclude the influence of a cross-over effect of fatigue between legs in our current results. Nevertheless, the variation in discharge rate pre and post fatigue in the non-paretic leg was similar to previous reports on healthy individuals (30), so the problem, even if not avoidable, was likely of moderate effect.

In conclusion, our data are clinically relevant as we demonstrate a relationship between task duration and leg function. Muscle fatigability is functionally relevant as many activities of daily living require the intermittent or sustained generation of sub-maximal forces. Taken together, our findings underscore the need to make clinical measures of fatigability of functionally relevant muscles to fully assess motor capability post stroke.

## Data Availability Statement

The datasets generated for this study are available on request to the corresponding author.

## Ethics Statement

The studies involving human participants were reviewed and approved by Institutional Review Board at Marquette University (HR-2753). The patients/participants provided their written informed consent to participate in this study.

## Author Contributions

AH, SH, and FN conceived and designed the research program. AH provided lab settings. KB, CB, JN, and AH conducted the experiments. FN and AH planned, carried out the signal analysis and the statistics, and wrote the first draft of the manuscript. All authors discussed the results and contributed to the final manuscript.

## Conflict of Interest

The authors declare that the research was conducted in the absence of any commercial or financial relationships that could be construed as a potential conflict of interest.

## Abbreviations

EMG: electromyography
MVC: maximal voluntary contractions
RMS: root mean square.
